# A hypomorphic inherited pathogenic variant in *DDX3X* causes male intellectual disability with additional neurodevelopmental and neurodegenerative features

**DOI:** 10.1186/s40246-018-0141-y

**Published:** 2018-03-01

**Authors:** Georgios Kellaris, Kamal Khan, Shahid M. Baig, I-Chun Tsai, Francisca Millan Zamora, Paul Ruggieri, Marvin R. Natowicz, Nicholas Katsanis

**Affiliations:** 1 Center for Human Disease Modeling, Duke University, 300 North Duke Street, Durham, NC 27701 USA; 2 Department of Medical Genetics, University of Athens Medical School, Aghia Sophia Children’s Hospital, 11527 Athens, Greece; 3 Human Molecular Genetics Laboratory, Health Biotechnology Division, National Institute for Biotechnology and Genetic Engineering (NIBGE), Faisalabad, 38000 Pakistan; 4 Imaging Institute, Cleveland Clinic, 9500 Euclid Avenue, Cleveland, OH 44195 USA; 5 Pathology and Laboratory Medicine and Genomic Medicine Institutes, Cleveland Clinic, Cleveland, OH 44195 USA; 6 GeneDx, 207 Perry Parkway, Gaithersburg, MD 20877 USA

## Abstract

**Background:**

Intellectual disability (ID) is a common condition with a population prevalence frequency of 1–3% and an enrichment for males, driven in part by the contribution of mutant alleles on the X-chromosome. Among the more than 500 genes associated with ID, *DDX3X* represents an outlier in sex specificity. Nearly all reported pathogenic variants of *DDX3X* are de novo, affect mostly females, and appear to be loss of function variants, consistent with the hypothesis that haploinsufficiency at this locus on the X-chromosome is likely to be lethal in males.

**Results:**

We evaluated two male siblings with syndromic features characterized by mild-to-moderate ID and progressive spasticity. Quad-based whole-exome sequencing revealed a maternally inherited missense variant encoding p.R79K in *DDX3X* in both siblings and no other apparent pathogenic variants. We assessed its possible relevance to their phenotype using an established functional assay for DDX3X activity in zebrafish embryos and found that this allele causes a partial loss of DDX3X function and thus represents a hypomorphic variant.

**Conclusions:**

Our genetic and functional data suggest that partial loss of function of *DDX3X* can cause syndromic ID. The p.R79K allele affects a region of the protein outside the critical RNA helicase domain, offering a credible explanation for the observed retention of partial function, viability in hemizygous males, and lack of pathology in females. These findings expand the gender spectrum of pathology of this locus and suggest that analysis for *DDX3X* variants should be considered relevant for both males and females.

**Electronic supplementary material:**

The online version of this article (10.1186/s40246-018-0141-y) contains supplementary material, which is available to authorized users.

## Background

Intellectual disability (ID) is a common (estimated prevalence of 1–3% [1–3]), clinically variable phenotype, defined by impairment in both intellectual function and adaptive behavior, with onset during the developmental period [4, 5]. ID is clinically and etiologically heterogeneous, with varying disability of the two aspects of the condition [6]. In addition, ID can occur as an isolated trait but is accompanied frequently by one or more co-morbidities; these can include seizures or other neurological involvement [7], microcephaly or macrocephaly [8], dysmorphic features, and structural and/or functional abnormalities in organ systems other than the brain [9].

ID is more prevalent in males than females with a ratio of 1.3–1.4:1 [10], a gender bias partially reflective of the observation that numerous genes on the X-chromosome contribute to ID under an X-linked recessive paradigm [10]. Indeed, substantial gene discovery efforts have focused on male-enriched or male-specific cohorts as a means to accelerate the discovery of ID-associated genes and mutational mechanisms [10]. Genetic and genomic studies of individuals, families, and populations with ID have led to the identification of more than 500 mutated genes, with some estimates predicting that number to exceed 1000, possibly reflective of the large number of transcripts expressed in and necessary for the development and maintenance of the central nervous system [10, 11]. In several instances, female carriers are protected by virtue of functional mosaicism due to random X-inactivation or by X-inactivation that favors the wild-type allele [12]. Further phenotypic analyses have also led to the appreciation of subtle phenotypic defects under a dosage model, in which males manifest the extreme range of pathology and females are partially protected [13].

The *DDX3X* locus on Xp11.4 represents an exception to the male specificity paradigm. To date, *DDX3X* pathogenic variants have been reported in 35 females with ID [3, 14], all of which were de novo. As of September 2017, 52 cases have been deposited in Decipher: 49 females, all but one of whom have de novo alleles (the 50th was inherited from a mosaic father) and three males, two harbor de novo mutations and one whose mutation is of unknown origin [15]. Consistent with the intolerance of the locus to variation [3], there are no null alleles found in control populations of either gender and a concomitant depletion of missense variants as well [16].

Herein, we report our investigation of two adult male siblings with ID, born to parents of normal intelligence. Whole-exome sequencing (WES) identified a rare, non-synonymous, maternally transmitted variant in *DDX3X* as a likely driver of their phenotype. DDX3X is necessary for canonical *Wnt* signaling [17], an observation utilized previously to develop an in vivo ventralization assay in zebrafish embryos as a means to assess the pathogenic potential of alleles [3]. Using this assay, we found that the *DDX3X* variant results in reduced DDX3X function, reinforcing the genetic hypothesis. Together, our data support the notion that partial loss of function in *DDX3X* can lead to ID in males and reinforce a gradient of severity model that is dependent on the strength of the mutation as it pertains to residual protein function.

## Materials and methods

### Subjects

We studied a pedigree with two affected male siblings. Diagnostic testing of subjects was done as part of their clinical evaluations with informed consent from the mother and assent of the two cases. Research-based testing protocols were approved by the Cleveland Clinic Institutional Review Board.

### Whole-exome sequencing

WES was carried out at GeneDx, Inc. subsequent to informed consent of the parents and assent of the affected individuals. Using genomic DNA from the ID cases and their parents, the exonic regions and flanking splice junctions of the genome were captured using the Clinical Research Exome kit (Agilent Technologies, Santa Clara, CA). Massively parallel (NextGen) sequencing was done on an Illumina system with 100 bp paired-end reads. The percent coverage at 10X or greater in the two brothers and their two parents ranged from 97.14 to 97.49%; the younger brother had 97.18% coverage that was at least 10X and the older brother had 97.49% coverage at least at 10X. The mean coverage in the younger brother was 132X and in the older brother 197X. The mean coverage in the father and the mother was 134.29X and 222.05X, respectively.

### Data analysis

Reads were aligned to human genome build GRCh37/UCSC hg19 and were analyzed for sequence variants using a custom-developed analysis tool. Additional sequencing technology and variant interpretation protocols have been described [18]. The general assertion criteria for variant classification are available on the GeneDx ClinVar submission page (http://www.ncbi.nlm.nih.gov/clinvar/submitters/26957/). Variants of possible clinical significance relative to the phenotype of interest were confirmed by Sanger sequencing. Variants were named according to the following GenBank identifiers: *DDX3X* (NM_001356) and *SPG7* (NM_003119).

### In vivo complementation studies in zebrafish

We obtained a plasmid containing the wild-type (wt) human open reading frame (ORF) of *DDX3X* and *WNT3A* from Ultimate ORF Collection (LifeTechnologies; clone ID *DDX3X*: IOH13891; *WNT3A*: IOH80731). Both plasmids were sequence-confirmed and cloned into the pCS2+ vector using Gateway LR clonase II-mediated recombination (LifeTechnologies) as described [19]. For *WNT3A*, a stop codon was introduced by site-directed mutagenesis using primer (5′-ctgcaaggccgccaggcacTAGGGTGGGCGCGCCGA-3′ and its reverse complement). To generate the mutant constructs for the ID-associated variant c.236 G>A (p.R79K) along with the positive control female variant c.641T>C (p.I214T) and a negative control male variant c.898G>T (p.V300F), we conducted site-directed mutagenesis using primers (c.641T>C: 5′-CTATTCCTATTACCAAAGAGAAAAG-3′, c.898G>T: 5′CTAGAGTTCGTCCTTGCGTGTTTTATGGTGGTGCCGATATT-3′, and c.236 G>A G: 5′-TTGGATCTCGTAGTGATTCAAAAGGGAAGTCTAGCTTC-3′ and their reverse complements). We then generated capped mRNA from linearized wt-*DDX3X* and *WNT3A* pCS2+ constructs as well as for *DDX3X* variants with the mMessage mMachine SP6 kit (ThermoFisher). Injections were conducted with wt (ZDR) embryos, and resulting embryos were phenotyped at 48 hpf for eye phenotypes according to established criteria (class I: hypoplasia of the eye and class II: absence of one or both eyes; [3]. The mRNAs (wt-*WNT3A*, wt-*DDX3X*, and *DDX3X* variants) were injected in the yolk of the embryos at 1–4 cell stage as described [20]. We acquired lateral and dorsal images with an AZ100 florescent microscope (Nikon), digital sight black and white camera (Nikon), and NIS Elements software (Nikon) at × 4 magnification. We determined statistical differences between pairs of batches with an unpaired *t* test.

## Results

### Clinical evaluations

**Fig. 1:**
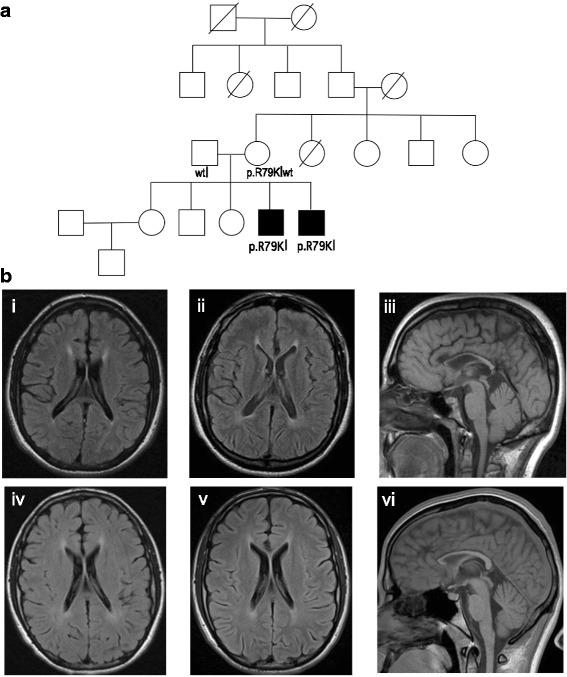
Brain MRI of both affected syndromic ID cases. **a** Family pedigree showing the two affected brothers and the family pedigree. **b** (i) Axial T2 FLAIR of sibling 1 at age 16 demonstrates mild enlargement of the lateral ventricles and mild confluent hyperintensity in the adjacent white matter. (ii) Axial T2 FLAIR of sibling 1 2 years later demonstrates interval enlargement of the lateral ventricles and mild progression of the confluent hyperintensity in the adjacent white matter suggesting progressive damage to the central white matter and volume loss. (iii) Sagittal T1 demonstrates generalized volume loss in the corpus callosum that is more prominent in the genu and anterior body, further supporting central white matter volume loss that is more severe anteriorly. (iv) Axial T2 FLAIR of sibling 2 at age 15 demonstrates mild enlargement of the lateral ventricles and mild, symmetric hyperintensity in the adjacent white matter, suggesting central white matter volume loss and gliosis. (v) Axial T2 FLAIR of sibling 2 2 years later demonstrates mild interval increase in size of the lateral ventricles suggesting mild progression of central white matter volume loss, but no significant change in the periventricular hyperintensity. (vi) Sagittal T1 image is also comparable in appearance to his sibling, with prominent volume loss in the corpus callosum that is more striking anteriorly

We evaluated two affected male siblings from a non-consanguineous family (Fig. 1; Table 1; for full clinical descriptions, see Additional file 1). The eldest affected, currently 29 years old, was diagnosed with ID, macrocephaly, dysarthria, progressive spastic paraparesis, and decreased lower extremity strength. He presented for clinical evaluation at 2 years of age; he had reduced speech output for age and was easily agitated and hyperactive. He walked at 15 months, said his first words at 1–2 years, talked in sentences at 3–4 years, and was toilet-trained at 3 years. Developmental assessment at 6.5 years showed nonverbal cognitive function and composite mental processing at 5 and 3 percentiles, respectively, and adaptive behavior functions ranging from 0.3 to 3%. At age 9.5 years, he showed verbal, performance, and full-scale IQ scores of 71, 80, and 73, respectively. Assessment of communication function showed receptive and expressive language function at about 0.5 and 0.04 percentiles, while adaptive behavior assessment showed communication, daily living, and socialization functions at 1, 2, and 10 percentiles, respectively.

**Table 1:** Summary of clinical findings in the two subjects. Human phenotype ontology (HPO) terms and codes are shown

Phenotype	HPO code	Case 1	Case 2
Intellectual disability or related neurodevelopmental disability	HP:0001256	+	+
Macrocephaly	HP:0000256	+	+
Dysarthria	HP:0001260	+	+
Tight heel cords		+	+
Progressive spastic paraparesis	HP:0007199	+	+
Tremor	HP:0002322	–	+
Hand weakness	HP:0030237	+	–
Proximal leg weakness	HP:0007340	+	+
Brain MRI with abnormal periventricular T2 intensity	HP:0002518	+	+
Ventriculomegaly	HP:0002119	+	+
Atrophy of corpus callosum, esp. the genu and anterior body of corpus callosum	HP:0006989	+	+
Increased cerebrospinal fluid alanine level	n/a	+	+

*n/a* not applicable

At 14 years of age, he developed a stiff gait. Exam at 15 years showed an acquired macrocephaly, an asymmetric spastic gait, lower extremity hyperreflexia, and tight heel cords. He began treatment with oral baclofen for spasticity at 17 years of age. He had an adjusted curriculum during high school, graduated at 19 years, and has worked part-time afterwards in a supported workplace. His gait slowly worsened and he was last able to ambulate independently at 21 years. On neurological exam at 23 years, there was a slow, spastic gait, decreased lower extremity strength, especially with hip flexion, markedly increased the tone of the lower extremities, lower extremity hyperreflexia, and slowed fine finger and rapid alternating movements. At 29 years of age, he has progressive lower extremity and hand weakness and decreased fine motor function.

Brain MRI scan at 16 years of age (Fig. 1i) showed mildly prominent lateral ventricles and cortical sulci, atrophy of the entire corpus callosum that was accentuated in the anterior body and genu, and mild, symmetric, confluent T2-hyperintensity in the supratentorial periventricular white matter. The follow-up study 2 years later demonstrated further interval enlargement of the lateral ventricles and periventricular white matter T2-hyperintensity, suggesting progression of central white matter volume loss. Myelination was otherwise within normal limits (Fig. 1ii). Cerebrospinal fluid analyses at 19 years showed a normal cell count, CSF glucose, and protein levels and normal CSF IgG synthesis and index. Metabolic testing included increased CSF alanine and intermittent mildly increased urinary lactate (Additional file 2: Table S1).

The younger affected brother, 25 years old, has progressive spastic paraparesis and tremor and learning disability/mixed expressive-receptive language disorder (Fig. 1; Table 1). The first concern about his neurodevelopmental status was when he was about 2 years old and had mildly delayed speech development; he spoke in sentences at 3 years and began speech therapy at that time. He developed an intentional hand tremor at about 6 years that worsened over time. Developmental assessment at 5.6 years showed normal development in all areas except for mild receptive and expressive language delay. Re-evaluation at 6 years showed receptive and expressive language skills at 39 and 16 percentiles, with weaknesses in auditory memory, interpreting directions, and categorizing words. Assessment at 7 years showed verbal, performance, and full-scale IQ scores of 84, 81, and 81, respectively. Academic achievement testing showed basic reading, reading comprehension, mathematics calculation, mathematics reasoning, and written expression at 1, 5, 16, 9, and 23 percentiles, respectively; receptive and expressive language tested at 37 and 16 percentiles. He did not have significant behavioral issues.

Examination at 15 years old, for worsening hand tremors, showed a non-dysmorphic male with lower extremity hyperreflexia and tremor; the gait and motor tone were normal. At 17 years, he was noted to have a mild spastic gait and bilateral Babinski signs. At 18 years, he was noted to have a worsening asymmetric spastic paretic gait and bilateral hip flexor weakness. Schooling involved an adjusted curriculum with training in service occupations, and he graduated from high school at 18 years of age, subsequently working part-time and requiring supports in complex daily living tasks.

His diagnostic evaluation included an abnormal brain MRI scans at 15, 16, and 17 years of age showing mild, diffuse prominence of the cortical sulci suggesting mild cortical volume loss and mild enlargement of the lateral ventricles, generalized volume loss in the corpus callosum that was more prominent in the genu and anterior body, and mild symmetric confluent periventricular T2-hyperintensity within the supratentorial periventricular white matter suggesting central white matter volume loss that was more prominent in the frontal lobes (Fig. 1iv–vi). There was also slight further enlargement of the lateral ventricles without significant change in the T2-hyperintensity or the corpus callosum over the 2-year period. The MRI of the entire spinal cord was normal in caliber and signal intensity. CSF analyses showed normal cellular CSF and normal levels of CSF glucose and protein, normal CSF IgG synthesis and IgG index, normal levels of CSF lactate and pyruvate, and increased CSF alanine. Plasma amino acid analyses consistently showed mildly moderately increased levels of alanine and, sometimes, increased levels of proline and glycine (Additional file 2: Table S1).

### Genomic analysis

**Fig. 2:**
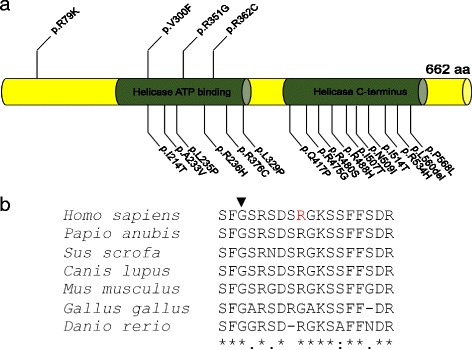
Discovery of a maternally transmitted *DDX3X* variant in male ID. **a** Protein sequence alignment of DDX3X across vertebrate species; the mutated residue is shown by arrow. **b** Location of all functionally tested amino acid substitutions in DDX3X. Reported male alleles (top); alleles found in females (bottom). The helicase ATP-binding domain and a helicase C-terminal domain are also shown (green)

A host of known genetic and metabolic etiologies of ID and of spasticity were ruled-out in the two cases (Additional file 2: Table S1). Consequently, given that both parents are of normal intelligence, we undertook quad-based WES analysis hypothesizing either a recessive or an X-linked paradigm of disease inheritance. Under a rare variant hypothesis, the WES data were evaluated for alleles shared among the two affected siblings that are rare (minor allele frequency < 1%) and affect coding residues or canonical splice sites. We identified several variants of potential interest (Additional file 3: Table S2), but only a single, maternally inherited variant fulfilled our stringent criteria: a c.236G>A change encoding p.R79K in *DDX3X* was identified (Fig. 2a, b). The *DDX3X* locus has been implicated previously in ID and, sometimes, other features in females; however, nearly all published variants described to date are de novo, which has led to the hypothesis that haploinsufficiency of *DDX3X* in males might be incompatible with life [3]. Nonetheless, multiple sequence alignment across vertebrates showed the p.R79 position to be conserved (Fig. 2b). Prediction algorithms were discordant: PolyPhen2 [21] predicted the allele to be benign, whereas MutationTaster [22] predicted it to be deleterious. Overall, 11 variant prediction algorithms predict this variant to be damaging to the structure/function of DDX3X out of a total of 21 in silico assessments [23], http://159.226.67.237/sun/varcards/welcome/index). The variant was found only once in heterozygosity, in a female, in > 170,000 control exomes [16]. We note that, in contrast to the majority of described *DDX3X* variants that lie in the helicase domain, the p.R79K variant maps proximal to that region (Fig. 2b). Together, these data raised the hypothesis that, in contrast to previously reported alleles in *DDX3X* that abrogate protein function, p.R79K might be a hypomorph, which could explain its transmission and the phenotypic outcome in this kindred.

### Functional testing

**Fig. 3:**
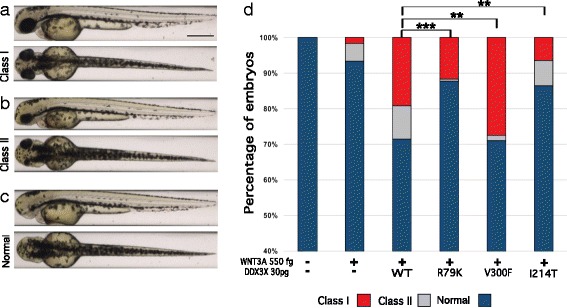
Functional testing of *DDX3X* variants. **a**–**c** Representative lateral images of zebrafish embryos at 2 dpf that are either uninjected (**a**) or injected with human *WNT3A* without (**b**) or with (**c**) human *DDX3X* show a range of ventralized phenotypes. These were scored according to established criteria as normal, class I, or class II ventralization. No injection condition resulted in severe ventralization (class III or IV) [3]. **d** *DDX3X* variants were tested for their effect on increasing *WNT3A*-mediated ventralization using 550 fg of *WNT3A* mRNA and 30 pg of *DDX3X* mRNA per embryo. *P* values: < 0.0001 (four asterisks); 0.0001 to 0.001 (three asterisks); 0.001 to 0.01 (two asterisks); 0.01 to 0.05 (one asterisk); ≥ 0.05 not significant (ns)

We and others have shown that expression of human *DDX3X* mRNA exacerbates the ventralization phenotype induced by the expression of femtogram (fg) quantities of the canonical Wnt ligand *WNT3A* in zebrafish embryos [3]. This phenotype can be binned into two phenotypic classes (class I and class II; Fig. 3a–c) based on previously established and validated morphometric criteria [24]. Using this model system, we tested the functionality of missense *DDX3X* alleles discovered in ID patients and found that DNA sequence variants predicted to cause loss of function of *DDX3X* and which were associated with ID failed to exacerbate WNT3A-driven pathology, whereas benign alleles were indistinguishable in their activity and promoted significant Wnt-dependent pathology [3]. Here, we utilized this assay to test the activity of the p.R79K allele. First, we optimized the in vivo complementation assays to the *ZDR* wt genetic background by injecting progressively increasing doses of wt-*WNT3A*; we found that 550 fg of mRNA was sufficient to induce a modest phenotype (~ 8–15% affected embryos, *n* = 50–100 embryos per clutch, replicated; Fig. 3, Additional file 4: Figure S1, Additional file 5: Figure S2A). On this background sensitization with *WNT3A*, we titrated the amount of human *DDX3X* mRNA required to generate pathology. We found a dose-dependent effect that was maximal at 30 pg mRNA (Additional file 4: Figure S1). Therefore, we selected the 500 fg/30 pg mRNA doses to test the effect of the discovered allele. Next, we co-injected *WNT3A*-sensitized *ZDR* embryos with wt *DDX3X*, *DDX3X* encoding the candidate pathogenic allele p.R79K, the known benign allele p.V300F, and the known pathogenic allele p.I214T. Upon blind scoring to injection cocktail, both the positive and negative controls scored as expected: mRNA encoding p.300F was indistinguishable from wt, whereas p.214T failed to induce any significant pathology exceeding that of *WNT3A* alone, consistent with a null (or near null) allele. The candidate mutation of interest, p.R79K, scored intermediate, with the extent of pathology of the resulting embryos significantly different from both wt and null *DDX3X* (*p* < 0.0008 and *p* < 0.01 respectively; Fig. 3d). In contrast, expression of either wt or mutant *DDX3X* mRNA alone did not induce any appreciable pathologies, likely excluding any dominant negative mechanisms (Additional file 5: Figure S2B). Together with the human genetics and evolutionary conservation, these data suggest that the discovered allele is a likely hypomorph.

## Discussion

Here, we describe the clinical phenotypes of two adult males who have a rare, maternally inherited missense allele of *DDX3X*, a major ID locus that to date has been associated with pathology almost exclusively in females. Both genetics and functional testing support a partial loss of function disease transmission. This notion is consistent with the observation that the mother who transmitted the p.R79K is asymptomatic and that the two affected males have significant neurodevelopmental pathology but have survived. Although the formal possibility remains that the mother might exhibit sub-clinical neuroanatomical findings, careful re-examination revealed no overt neurological concerns. In addition to this notion, it would be important to identify the genotype for the maternal uncle and maternal grandfather, but DNA samples from the individuals were not available. This hypothesis is also supported by recent work in the mouse revealing essential roles of *Ddx3x* in placentation and embryogenesis and the differential phenotypic impacts of inheriting a paternal vs. maternal null allele [25]. Non-lethality of the p.R79K variant in our cases may be due to the maternal, as opposed to paternal, inheritance of the variant. Non-lethality of the p.R79K variant might also be because the location of the amino acid substitution is outside of the critical RNA helicase domain. The DDX3X protein has been investigated extensively [26], but the region of the protein surrounding amino acid residue 79 has only been studied sparingly, although site-directed mutagenesis of several nearby residues resulted in functional impacts [27, 28]. Finally, non-lethality of the p.R79K variant might be explained on the basis of the expected moderate biophysical impact on the structure of DDX3X consequent to the conservative nature of an arginine to lysine substitution.

There is substantial clinical and neuroradiologic overlap between the phenotype in our two cases and those of the females and few males described to date. The most common traits described thus far include ID, microcephaly, hypotonia, movement disorder and/or spasticity, ventricular enlargement, and hypoplasia of the corpus callosum; the subjects reported here have ID, spasticity, ventricular enlargement, and an attenuated corpus callosum. Our two cases also present with features that are distinct from those of other cases with variants in *DDX3X* reported to date; these include progressive spasticity with loss of independent ambulation and, consistent with their clinical courses, evidence of a progressive brain central white matter process including atrophy of the corpus callosum, not hypoplasia. Long-term follow-up of the previously reported cases, some of whom were young children, will be important to determine if any of the neurological findings or developmental disabilities are progressive and if there are ongoing brain white matter changes.

It is also possible that a second clinical process that is distinct from DDX3X may be present in the two subjects reported here. In this regard, both subjects have macrocephaly, as does their father and one sister although the father and the subjects’ siblings have clinical phenotypes that are otherwise disparate from the clinical findings in the cases with ID. The finding of increased levels of CSF alanine in both cases and of increased plasma alanine in one case and increased urinary lactate in the other is notable and suggests a disturbance of mitochondrial bioenergetics. No evidence of a primary mitochondrial cytopathy was found, despite considerable investigation, and the relationship of these results to the variant of *DDX3X* is uncertain. In addition, the existence of a clinical process that is/was present in only one of the subjects is likely in view of the variability in neurodevelopmental phenotype between the subjects, although the basis of this process is unknown. Given the reported spasticity in this pedigree, we did evaluate known spastic paraplegia genes for candidate pathogenic variants. We detected a maternally inherited heterozygous variant in *SPG7* in one of the subjects that has been reported to be pathogenic in patients with recessive spastic paraplegia [29]. Although this allele is by itself unable to explain the observed spasticity in our patient, it is possible that it contributes to the phenotype, possibly in epistasis with *DDX3X*; the same might be true for some of the other ultra-rare alleles discovered in this family (Additional file 3: Table S2). For example, it is intriguing that the older brother with the more extensive pathology is also hemizygous for candidate pathogenic variant in *ARGHGAP4*, a gene whose product is required for neuronal migration and which has been reported to be mutated in some patients with ID [30–32]. However, both formal functional testing and delineation of additional patients will be necessary to test this hypothesis.

## Conclusions

Overall, our findings suggest that mutational analysis of *DDX3X* in either genetic testing panels or by whole-exome/genome sequencing should not be limited, by testing or interpretation of data, to females. The severity of most discovered *DDX3X* alleles remains consistent with a model of total haploinsufficiency incompatible with life in males. Further studies in expanded cohorts will illuminate this proposed phenotype-genotype correlation further.

## Additional files

Additional file 1: Detailed Clinical report. (DOCX 23 kb)

Additional file 2: **Table S1.** Clinical Laboratory Diagnostic Evaluations. (DOCX 16 kb)

Additional file 3: **Table S2.** Sequence variants identified according to mode of inheritance. (DOCX 16 kb)

Additional file 4: **Figure S1.** Co-injection of *DDX3X* and *WNT3A* mRNA produces dose-dependent changes in ventralization. Zebrafish embryos were injected with 550 fg *WNT3A* mRNA without or with wt *DDX3X* mRNA at varying doses. Embryos were scored at 48 h post fertilization for degree of ventralization according to described objective criteria. *P* value: < 0.0001, (****); 0.0001 to 0.001, (***); 0.001 to 0.01, (**); 0.01 to 0.05, (*); ≥ 0.05, not significant (ns). (PDF 1597 kb)

Additional file 5: **Figure S2.** Dose-response effect of WNT3A and overexpression of DDX3X variants does not produce alterations in Wnt signaling. (**A**) Injection of WNT3A produces dose-dependent changes in ventralization. Embryos were scored at 48 h post fertilization for degree of ventralization. (**B**) Zebrafish embryos were injected with 100 pg of DDX3X mRNA containing either the wild-type sequence or the non-synonymous variants found in the affected individuals. At 36 h, post-fertilization (hpf) the embryos were phenotyped; no abnormalities could be appreciated. (PDF 1438 kb)
