# Supplementary material for: A hypomorphic inherited pathogenic variant in DDX3X causes male intellectual disability with additional neurodevelopmental and neurodegenerative features
Source: Hum Genomics. 2018 Mar 1;12:11. doi: 10.1186/s40246-018-0141-y (PMC5831694; doi:10.1186/s40246-018-0141-y)
Supplement: Supplementary file 1 — Detailed Clinical report. (DOCX 23 kb) [file 40246_2018_141_MOESM1_ESM.docx]

**­­­Supplemental Note 1. Detailed Clinical report**

**Patient 1.**

Patient 1 was born at 38.5 weeks gestation to a healthy 30-year-old mother after an uncomplicated pregnancy, labor and delivery; the birth weight and length were 9 lbs and 19 inches, respectively. There were no complications of the perinatal or neonatal periods. He presented for clinical evaluation at about 2 years of age when it was apparent that he had reduced speech output for age and was easily agitated and hyperactive. He walked at 15 months, said his first words at 1-2 years, talked in sentences at 3-4 years, and was toilet-trained at 3 years. There was a history of multiple bouts of otitis media and strabismus. At 65 months of age, developmental assessment showed a shortened attention span and delayed receptive and expressive language skills. The Peabody Picture Vocabulary Test showed verbal comprehension and expressive language function at <1%; Conner’s rating scales indicated T scores of >70 with respect to conduct, learning, and impulsivity-hyperactivity. Behavioral issues at that time included hyperactivity, temper outbursts and intermittent aggressiveness. He began receiving speech and language therapy; special education services commenced shortly thereafter.

Developmental assessment at 6.5 years showed nonverbal cognitive function and composite mental processing at 5 and 3 percentiles, respectively, using the Kaufman Assessment Battery. Language assessment using the CELF instrument showed receptive and expressive language function at 3 and 0.1 percentiles, respectively, and the Vineland Adaptive Behavior Scales showed adaptive behavior functions ranging from 0.3 to 3 percentiles.

He had a several day history of fever and increased cerebrospinal fluid (CSF) leukocytes and presumed viral encephalitis at 9 years from which he had a complete recovery. At 9.5 years testing, using Wechsler Intelligence Scale for Children (WISC)-III, showed verbal, performance and full scale IQ scores of 71, 80 and 73, respectively, and academic achievement, using the Woodcock-Johnson instrument, showed scores ranging from 0.3 to 7 percentiles with greatest difficulties in math concepts and calculation. Assessment of communication function using the CELF instrument showed receptive and expressive language function at 0.5 and 0.04 percentiles. Adaptive behavior assessment, using the Vineland instrument, showed communication, daily living and socialization functions at 1, 2 and 10 percentiles, respectively. At 12.5 years testing, using WISC-III, showed verbal, performance and full-scale IQ scores of 56, 72 and 61, respectively, and academic achievement, assessed with the Wechsler Individual Achievement Test instrument, ranged from <1-3 percentiles, with areas of greatest difficulty including reading comprehension and relative strength in written expression. Receptive and expressive language function both tested at about 0.04 percentile.

At 14 years of age he developed a stiff gait. Exam at 15 years was notable for an acquired macrocephaly, an asymmetric spastic gait, mild lower extremity hyperreflexia and tight heel cords. Testing at nearly 16 years, using WISC-IV, showed a full-scale IQ of 50 and academic achievement, assessed using WIAT, ranged from <0.1 to 1 percentiles, with greatest difficulties in reading comprehension and mathematical reasoning. He began treatment with oral Baclofen for his spasticity just before 17 years of age. His gait slowly worsened and he was last able to ambulate independently at 21 years. Later, he needed a walker and, sometimes, a scooter. He had an adjusted curriculum during high school, graduated at 19 years, and has worked part-time afterwards in a supported workplace. He received a Baclofen pump at 22 years of age. Exam at 23 years showed a non-dysmorphic male with head circumference 60.6 cm (>99%; +3.84 SD), height 167 cm (9%; -1.33 SD) and an unremarkable general physical exam apart from tight heel cords. On neurological exam, he spoke in full and grammatically correct sentences that were slowly articulated with mild dysarthria and slowed processing of verbally presented information. There was a slow, spastic gait, decreased lower extremity strength especially with hip flexion, markedly increased tone of the lower extremities, lower extremity hyperreflexia, and slowed fine finger and rapid alternating movements. The thumbs were not adducted, there were no involuntary movements, and the sensory exam was normal. By 25 years of age, he was no longer able to ambulate independently but could ambulate short distances with a walker. There was mild decline of fine motor function with new difficulty grasping eating utensils. A dilated eye exam at 28 years was normal. At 29 years of age, he has progressive lower extremity and hand weakness and decreased fine motor function. There is no history of seizures, syncope, involuntary movements, dysphagia, problems with day or night visual acuity, photophobia, hearing dysfunction, bladder or bowel dysfunction, temperature dysregulation, headache, or sleep dysfunction and no current behavioral concerns.

His diagnostic evaluation includes abnormal brain MRI scan at 16 years of age (Figure 1B) showing mildly prominent lateral ventricles and cortical sulci, atrophy of the entire corpus callosum that was accentuated in the anterior body and genu, and mild, symmetric, confluent T2-hyperintensity in the supratentorial periventricular white matter. The follow-up study two years later demonstrated further interval enlargement of the lateral ventricles and periventricular white matter T2-hyperintensity, suggesting progression of central white matter volume loss (Fig S1). The deep gray matter structures, brainstem, cerebellum and cervical spinal cord were normal in appearance. The myelination was otherwise within normal limits for age and no distinct intracranial developmental abnormalities were noted. Cerebrospinal fluid analyses at 19 years showed a normal cell count, CSF glucose and protein levels, and normal CSF IgG synthesis and index. Metabolic testing included increased CSF alanine at 42 µM (NL: 21-35 µM) and intermittent mildly increased urinary lactate and the following normal levels: serum creatine kinase, biotinidase acivity, folate, methymalonate, homocysteine, 3-methylglutaconate and copper; blood lactate, pyruvate and ammonia; plasma amino acids and acylcarnitines; serum very long chain fatty acids and phytanic and pristanic acids; leukocyte lysosomal enzyme activities; urinary acylglycines, guanidinoacetate and creatine; urinary oligosaccharides and sialic acid; fibroblast lactate and pyruvate; and fibroblast mitochondrial respiratory chain enzyme activities and pyruvate dehydrogenase (Supplementary Table 1). Normal genetic testing included: a routine blood lymphocyte cytogenetic analysis; oligonucleotide-based chromosomal microarray analysis; negative testing for pathologic nucleotide repeat expansion of *FMR1*; and DNA sequencing of *L1CAM* and of *SPG11* (Supplementary Table 1). WES showed hemizygosity for a maternally inherited variant of *DDX3X* c.236 G>A (p.R79K) and heterozygosity for a maternally inherited variant of *SPG7* c.1529 C>T (p.A510V); no pathogenic variants of the mitochondrial genome were identified.

**Patient 2.**

The younger affected brother, 25 years old, has progressive spastic paraparesis, tremor and learning disability/mixed expressive-receptive language disorder (Figure 1; Table 1). He was born at 36.5 weeks gestation to a 34 year old mother whose pregnancy was complicated by gestational diabetes. The labor and vaginal delivery were unremarkable; the birth weight was 7 lbs 9 oz. The perinatal and neonatal periods were unremarkable, as was the remainder of his infancy. He sat at 6 months and walked at 12 months. The first concern about his neurodevelopmental status was when he was about 2 years old and had mildly delayed speech development; he spoke in sentences at 3 years and began speech therapy at that time. He had a bout of viral encephalitis at 3.5 years and had headaches for at least 10 years afterwards. He developed an intentional hand tremor at about 6 years; this slowly worsened and was diagnosed as a benign familial tremor. He later needed to learn to write with his left hand since his right hand, the dominant hand, was more affected. Developmental assessment at 5.6 years showed normal development in all areas except for mild receptive and expressive language delays; evaluation using the CELF instrument showed receptive and expressive functions at 5 and 8 percentiles, respectively, and he began receiving speech/language therapy. Re-evaluation at 6 years showed receptive and expressive language skills at 39 and 16 percentiles, with weaknesses in auditory memory, interpreting directions and categorizing words. Assessment at 7 years with WISC-III showed verbal, performance and full-scale IQ scores of 84, 81 and 81, respectively, with verbal comprehension, perceptual organization, freedom from distraction and processing speed scores of 88, 86, 75 and 86, respectively. Academic achievement assessed with the Woodcock-Johnson instrument showed basic reading, reading comprehension, mathematics calculation, mathematics reasoning and written expression at 1, 5, 16, 9 and 23 percentiles, respectively, and language assessment with the CELF instrument showed average range receptive language and low average expressive language at 37 and 16 percentiles and continued weaknesses in auditory memory, interpreting directions and categorizing words. He was diagnosed with Specific Learning Disability. He learned slower than his peers during childhood and, especially, his teens. He did not have significant behavioral issues.  Assessment at 10 years with WISC-III showed verbal, performance and full-scale IQ scores of 91, 72 and 80, respectively, with verbal comprehension, perceptual organization, freedom from distraction and processing speed scores of 95, 77, 78 and 77, respectively. Academic achievement assessed with the Woodcock-Johnson instrument showed basic reading, reading comprehension, mathematics calculation, mathematics reasoning and written expression at 13, 8, 3, 48 and 4 percentiles, respectively, and language assessment with CELF showed average range receptive language and borderline expressive language at 12 and 3 percentiles and continued weaknesses in auditory memory and categorizing words. Evaluation also showed him to be significantly inattentive but not hyperactive. Assessment at 13 years showed a full-scale IQ of 74 with scores of 93, 84, 62 and 73 for verbal comprehension, perceptual reasoning, working memory and processing speed, respectively, on the WISC-IV with areas of strength included verbal associative learning, social comprehension and spatial reasoning and areas of difficulty included working memory and graphomotor speed. Language assessment showed receptive and expressive language functions at 14 and 3 percentiles.

Examination at 15 years old, for worsening hand tremors, showed a non-dysmorphic male with lower extremity hyperreflexia and tremor; the gait and motor tone were normal. At 16.5 years he was treated with Primidone for the tremor; this was discontinued at 20 years of age. At 17 years the head circumference was 56 cm (73%; +0.63 SD); he was noted to have a mild spastic gait and bilateral Babinski signs. At 18 years he was noted to have a worsening asymmetric spastic paretic gait and bilateral hip flexor weakness. Treatment with oral Baclofen was begun. Schooling involved an adjusted curriculum with training in service occupations and he graduated from high school at 18 years of age, subsequently working part-time and requiring supports in complex daily living tasks. Assessment at 19 years with WISC-III showed verbal, performance and full-scale IQ scores of 81, 80 and 79, respectively, with verbal comprehension, perceptual organization, working memory and processing speed scores of 88, 88, 69 and 68, respectively. He was diagnosed with a learning disability/mixed expressive-receptive language disorder in his 20s. Progressively worsening gait and imbalance led to occasional falls since about 20 years of age. Botox treatment was begun at 21 years old, which has been helpful. The head circumference then was 57.5 cm (95%; +1.67 SD) and height 176.6 cm (50%). There was slight dysarthria and worsening of the gait. At 22 years he was able to ambulate with bilateral AFOs and a cane; by 23 years he needed AFOs and a walker. There has been no history of seizures or concerns regarding visual acuity, photophobia, hearing difficulty, dysphagia, or bowel or bladder dysfunction.

His diagnostic evaluation included abnormal brain MRI scans at 15, 16 and 17 years of age (Figure 2B; Fig 1S) showing mild, diffuse prominence of the cortical sulci suggesting mild cortical volume loss and mild enlargement of the lateral ventricles, generalized volume loss in the corpus callosum that was more prominent in the genu and anterior body, and mild symmetric confluent periventricular T2-hyperintensity within the supratentorial periventricular white matter suggesting central white matter volume loss that was more prominent in the frontal lobe. There was also slight further enlargement of the lateral ventricles without significant change in the T2-hyperintensity or the corpus callosum over the two-year period. The MRI of the entire spinal cord was normal in caliber and signal intensity characteristics. CSF analyses showed a normocellular CSF with normal levels of CSF glucose and protein, normal CSF IgG synthesis and IgG index, absent CSF oligoclonal bands, normal levels of CSF lactate and pyruvate, and increased CSF alanine at 44 µM (NL: 21-35 µM). Metabolic testing showed normal levels of: serum folate, methylmalonate, homocysteine, uric acid, lactate, pyruvate, 3-methylglutaconate, ammonia, copper and ceruloplasmin; serum transferrin isoforms; plasma acylcarnitines, serum very long chain fatty acids and phytanic and pristanic acids; leukocyte lysosomal enzyme activities; urinary organic acids and acylglycines; urinary amino acids; and urinary oligosaccharides. Plasma amino acid analyses consistently showed mildly-moderately increased levels of alanine and, sometimes, increased levels of proline and glycine (Supplementary Table 1). Molecular genetic testing showed negative nucleotide repeat expansion mutation analyses of *ATXN1*, *ATXN2*, *ATXN3*, *CACNA1A*, *ATXN7*, *ATXN8*, *ATXN10*, *TBP*, *ATN1*, and *FXN*. DNA sequencing of the exons and intron/exon junctions was negative for *APTX*, *PRKC*, *SETX*, *SPTBN2*, *SIL1*, *TTPA*, *KCNC3,* *POLG1*, *ATL1*, *SPAST*, *NIPA1*, *WSHC5*, and *REEP1* and deletion analysis of *SPAST* (Supplementary Table 1). WES showed hemizygosity for a maternally inherited variant of *DDX3X* c.236 G>A (p.R79K); no pathogenic variants of the mitochondrial genome were identified.

The subjects have three siblings. First, a 35 year old sister has borderline macrocephaly (98%), chronic headaches, hypothyroidism, and complaint of "tight hamstrings and quads" but no spasticity or pathologic hyperreflexia noted on exam. A 33 year old brother is reportedly healthy. Finally, a 31 year old sister has mitral valve prolapse and endometriosis. Their 59 year old mother has osteoarthritis and mild spinal stenosis; she does not have neurological concerns and, specifically, does not have spasticity or vision problems. She has random X-chromosome inactivation as assessed by the androgen receptor-based assay using genomic DNA from peripheral blood lymphocytes. None of her siblings or parents have phenotypes analogous to those of her sons. The subjects’ 58 year old father is reportedly macrocephalic and has Behcet syndrome, a slight left hand tremor, a complaint of “tight muscles” and mild lumbar spinal stenosis. A paternal uncle of the two subjects had a neonatal death with hydrocephalus and a paternal cousin with a learning disability developed spasticity as a young adult and has a dizygotic twin sister with a learning disability.
