# Supplementary material for: A hypomorphic inherited pathogenic variant in DDX3X causes male intellectual disability with additional neurodevelopmental and neurodegenerative features
Source: Hum Genomics. 2018 Mar 1;12:11. doi: 10.1186/s40246-018-0141-y (PMC5831694; doi:10.1186/s40246-018-0141-y)
Supplement: Supplementary file 2 — Table S1. Clinical Laboratory Diagnostic Evaluations. (DOCX 16 kb) [file 40246_2018_141_MOESM2_ESM.docx]

| **Table S1: Clinical Laboratory Diagnostic Evaluations** | | |
| --- | --- | --- |
| TEST | CASE 1 | CASE 2 |
| CSF cell count | WNL | WNL |
| CSF glucose | WNL | WNL |
| CSF protein | WNL | WNL |
| CSF IgG synthesis & IgG index | WNL | WNL |
| CSF oligoclonal bands | negative | negative |
| CSF alanine | increased | increased |
| CSF lactate | WNL | WNL |
| CSF pyruvate |  | WNL |
| Serum uric acid | WNL | WNL |
| Serum folate | WNL | WNL |
| Serum homocysteine | WNL | WNL |
| Serum MMA | WNL | WNL |
| Blood lactate and pyruvate | WNL | WNL |
| Blood ammonia | WNL | WNL |
| Serum copper | WNL | WNL |
| Serum ceruloplasmin |  | WNL |
| Serum 3-methylglutaconate | WNL | WNL |
| Serum creatine kinase | WNL |  |
| Plasma amino acids | WNL | increased alanine |
| Plasma acylcarnitines | WNL | WNL |
| Serum biotinidase | WNL |  |
| Serum very long chain fatty acids | WNL | WNL |
| Serum phytanic and pristanic acids | WNL | WNL |
| Serum transferrin isoforms |  | WNL |
| WBC lysosomal enzymes | WNL | WNL |
| Urinary organic acids | intermittent increased lactate | WNL |
| Urinary acylglycines | WNL | WNL |
| Urinary amino acids |  | WNL |
| Urinary guanidinoacetate and creatine | WNL | WNL |
| Urinary oligosaccharides | WNL | WNL |
| Urinary sialic acid |  | WNL |
| Fibroblast lactate and pyruvate | WNL |  |
| Fibroblast respiratory chain enzymes | WNL |  |
| Fibroblast pyruvate dehydrogenase | WNL |  |
| Blood lymphocyte cytogenetic analysis | Normal 46,XY male |  |
| Oligonucleotide chromosomal microarray | negative |  |
| *FMR1* nucleotide repeat expansion | negative |  |
| DNA sequencing of *L1CAM* | negative |  |
| DNA sequencing of *SPG11* | negative |  |
| Nucleotide repeat expansion testing, ataxia panel^1^ | negative |  |
| DNA sequencing, ataxia panel^2^ | negative |  |
| Mitochondrial genome sequencing | negative | negative |
| Whole exome sequencing | see text | see text |
| ------------------------------------------------------------------------------------------------------------------------------------------------------------------------------------------------------------------------------- | | |
| ^1^Negative nucleotide repeat expansion mutation analyses of: *ATXN1, ATXN2, ATXN3, CACNA1A, ATXN7, ATXN8, ATXN10, TBP, ATN1* and *FXN*.  ^2^DNA sequencing was negative for *APTX, PRKC, SETX, SPTBN2, SIL1, TTPA, KCNC3, POLG1, ATL1, SPAST, NIPA1, WSHC5, and REEP1* as well as deletion analysis of *SPAST*.  WNL: Within normal limit | | |
