# Supplementary material for: A hypomorphic inherited pathogenic variant in DDX3X causes male intellectual disability with additional neurodevelopmental and neurodegenerative features
Source: Hum Genomics. 2018 Mar 1;12:11. doi: 10.1186/s40246-018-0141-y (PMC5831694; doi:10.1186/s40246-018-0141-y)
Supplement: Supplementary file 3 — Table S2. Sequence variants identified according to mode of inheritance. (DOCX 16 kb) [file 40246_2018_141_MOESM3_ESM.docx]

| **Table S2: Sequence variants identified according to mode of inheritance** | | | | | | | |
| --- | --- | --- | --- | --- | --- | --- | --- |
|  | **n** | **Gene** | **Resulting genes of interest** | **gnomAD**  **frequency** | **CADD**  **score** | **Provean** |  |
| ***De novo*** |  |  |  |  |  |  |  |
| Younger brother | 1 | ABCA7 | NM_019112:ex18:c.C2401T:p.P801S | 0 | 0.132 | -1.09 |  |
| Older brother | 0 |  |  |  |  |  |  |
| **X chromosome genes** |  |  |  |  |  |  |  |
| Both siblings | 3 | DDX3X | NM_001193416:ex4:c.G236A:p.R79K | 0 | 23.11 | -1.71 |  |
|  |  | LOC100288814 | NM_001195081:ex1:c.G298A:p.G100R | 0.0000319  (2 hemizygotes) | 24.8 |  |  |
|  |  | FGD1 | NM_004463:ex4:c.C935T:p.P312L | 0.0002913  (20 hemizygotes) | 12.18 | -3.26 |  |
| Younger brother | 0 |  |  |  |  |  |  |
| Older brother | 2 | TBC1D25 | NM_002536:ex6:c.G1946A:p.R649H | 0.00014990  (15 hemizygotes) | 24.7 | -2.64 |  |
|  |  | ARHGAP4 | NM_001164741:ex18:c.C2197T:p.R733W | 0.00014273  (13 hemizygotes) | 19.32 | -2.87 |  |
| **Homozygous** |  |  |  |  |  |  |  |
| Both siblings | 0 |  |  |  |  |  |  |
| Younger brother | 0 |  |  |  |  |  |  |
| Older brother | 0 |  |  |  |  |  |  |
| **Compound Heterozygous** |  |  |  |  |  |  |  |
| Both siblings | 0 |  |  |  |  |  |  |
| Younger sibling | 1 | GTPBP4 | NM_012341:ex4:c.G426C:p.K142N | 0.00064642 (no homozygotes) | 20.4 | -4.57 |  |
|  |  |  | NM_012341:ex5:c.C488A:p.T163N | 0.00000406 | 22.3 | -1.32 |  |
