# Supplementary figures and images for: A hypomorphic inherited pathogenic variant in DDX3X causes male intellectual disability with additional neurodevelopmental and neurodegenerative features
Source: Hum Genomics. 2018 Mar 1;12:11. doi: 10.1186/s40246-018-0141-y (PMC5831694; doi:10.1186/s40246-018-0141-y)

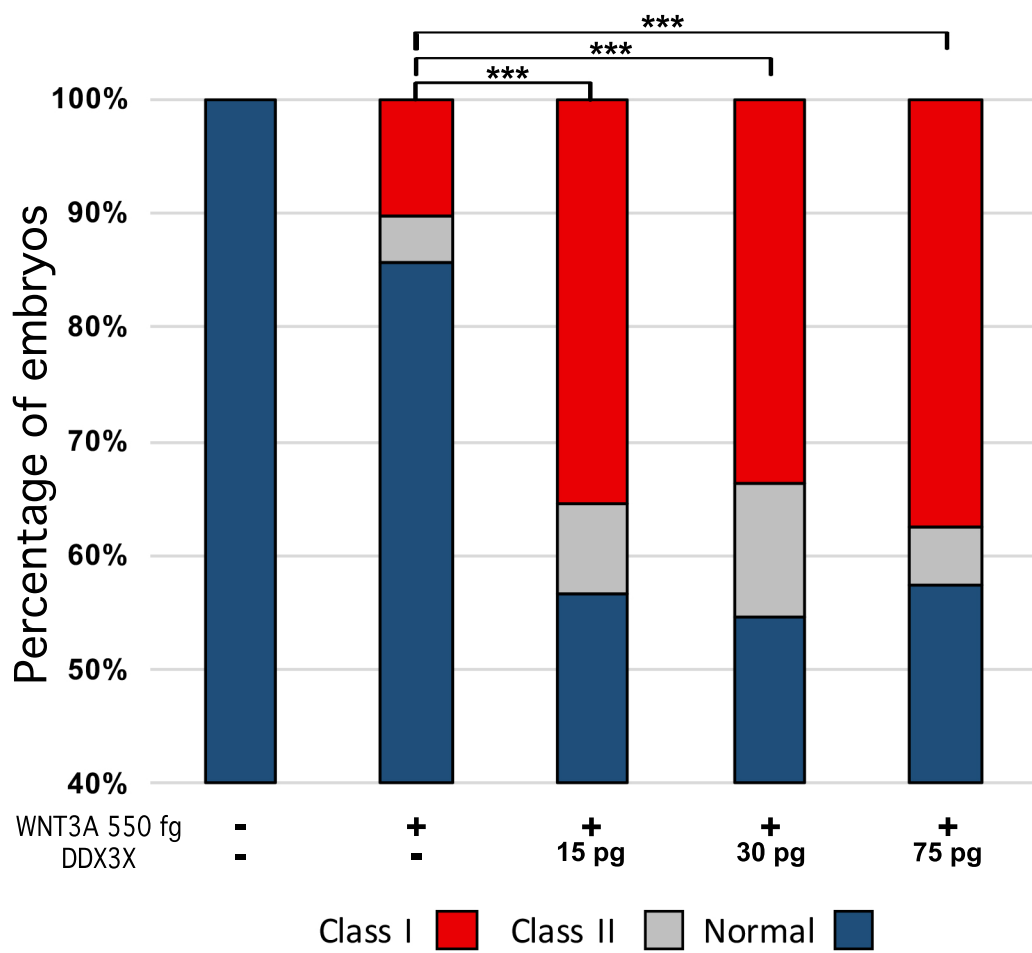

Supplemental Figure 1

Supplement: Supplementary file 4 — Figure S1. Co-injection of DDX3X and WNT3A mRNA produces dose-dependent changes in ventralization. Zebrafish embryos were injected with 550 fg WNT3A mRNA without or with wt DDX3X mRNA at varying doses. Embryos were scored at 48 h post fertilization for degree of ventralization according to described objective criteria. P value: < 0.0001, (****); 0.0001 to 0.001, (***); 0.001 to 0.01, (**); 0.01 to 0.05, (*); ≥ 0.05, not significant (ns). (PDF 1597 kb) [file 40246_2018_141_MOESM4_ESM.pdf]

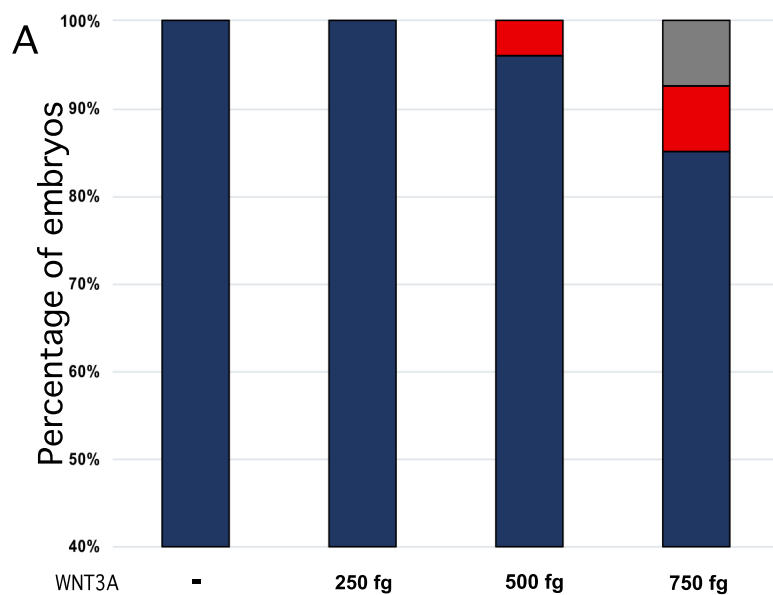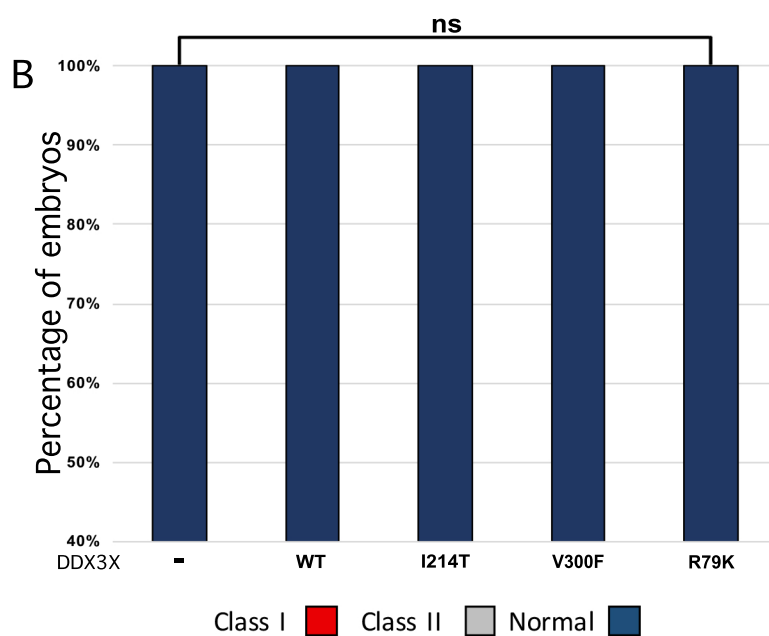

Supplement: Supplementary file 5 — Figure S2. Dose-response effect of WNT3A and overexpression of DDX3X variants does not produce alterations in Wnt signaling. (A) Injection of WNT3A produces dose-dependent changes in ventralization. Embryos were scored at 48 h post fertilization for degree of ventralization. (B) Zebrafish embryos were injected with 100 pg of DDX3X mRNA containing either the wild-type sequence or the non-synonymous variants found in the affected individuals. At 36 h, post-fertilization (hpf) the embryos were phenotyped; no abnormalities could be appreciated. (PDF 1438 kb) [file 40246_2018_141_MOESM5_ESM.pdf]
